# Learning About and Living With Toxicity: A Qualitative Study of Patients Receiving Immune Checkpoint Inhibitors For Melanoma or Lung Cancer and Their Caregivers

**DOI:** 10.21203/rs.3.rs-4576328/v1

**Published:** 2024-07-04

**Authors:** Ayo Samuel Falade, Mary C. Boulanger, Kelly Hsu, Roshni Sarathy, Riley Fadden, Kerry L Reynolds, Lara Traeger, Jennifer S. Temel, Joseph A. Greer, Laura A. Petrillo

**Affiliations:** Department of Medicine, Massachusetts General Brigham Salem Hospital; Division of Hematology and Oncology, Department of Medicine, Massachusetts General Hospital Cancer Center; Donald & Barbara Zucker School of Medicine at Hofstra/Northwell; Division of Palliative Care and Geriatrics, Department of Medicine, Massachusetts General Hospital Cancer Center; Division of Hematology and Oncology, Department of Medicine, Massachusetts General Hospital Cancer Center; Division of Hematology and Oncology, Department of Medicine, Massachusetts General Hospital Cancer Center; Division of Hematology and Oncology, Department of Medicine, Massachusetts General Hospital Cancer Center; Division of Hematology and Oncology, Department of Medicine, Massachusetts General Hospital Cancer Center; Department of Psychiatry, Massachusetts General Hospital; Division of Palliative Care and Geriatrics, Department of Medicine, Massachusetts General Hospital Cancer Center

## Abstract

**Background/Objective:**

Immune checkpoint inhibitors (ICIs) have revolutionized treatment for melanoma and lung cancer and are in widespread use. This study aims to describe how patients and caregivers learn about ICI toxicities and their perceptions and experiences of toxicity.

**Methods:**

We conducted a qualitative study of 42 patients with advanced non-small cell lung cancer (NSCLC; n = 16) or melanoma (n = 26) who were initiating or discontinuing an ICI and their caregivers (n = 9). We conducted in-depth interviews to explore patients’ and caregivers’ experiences learning about and living with ICI side effects. We audio-recorded the first oncology visit after enrollment. We used a framework approach to code interview and visit transcripts and synthesized codes into themes.

**Results:**

The median age of patients was 67; 68% were male. Themes of participant interviews and clinician-patient dialogue included: i) Patients initiating an ICI received extensive information about side effects, which some patients found overwhelming or scary and difficult to absorb; ii) Patients who were deterred by fear of toxicity ultimately proceeded with treatment because of oncologist encouragement or the sense of no alternative; iii) participants found hope in the association between toxicity and ICI efficacy; iv) caregivers helped patients navigate the deluge of information and uncertainty related to ICIs. Participants suggested ways to improve ICI side effect education, such as incorporating patient stories.

**Conclusion:**

Patients perceived that ICI toxicity counseling was overwhelming yet were encouraged by oncologists’ reassurance that serious side effects were manageable and by the framing of toxicity as a sign of efficacy. We identified opportunities to improve communication of ICI risks and benefits.

## Introduction

Immune checkpoint inhibitors (ICIs) have recently emerged as a paradigm-shifting treatment option for patients with melanoma and non-small cell lung cancer (NSCLC), offering hope and improved survival (1–5). However, in addition to activating the immune system to recognize cancer cells, ICIs can also cause novel toxicities that negatively impact patients’ quality of life (6, 7). Patient awareness of the possible risks of ICIs is important for several reasons. Toxicity in patients, especially those with reduced functional status or advanced age, may result in negative outcomes such as hospitalization or the need for immunosuppressive treatments that introduce new risks (8). Those with autoimmune disease may experience worsening of chronic conditions (9, 10). For all patients, prompt recognition and treatment of toxicities minimizes their risk and increases patients’ likelihood of continuing treatment (11). Thus, it is especially important for patients receiving ICIs to be informed of their risks and benefits given novel toxicities that require accurate expectations and vigilance.

Prior studies have demonstrated patient misperceptions of ICI risks and benefits (12–14). Patients generally have favorable perceptions of immunotherapy and its ability to allow them to continue everyday life, particularly in comparison to chemotherapy (15). There has been increased use of ICIs in the last days of life, likely due to their perceived tolerability and the hope for a “Lazarus effect”; however, end-of-life immunotherapy is associated with more intensive and less comfort-focused end-of-life care (16–22). Although an increasing number of patients across many cancer types are now receiving immunotherapy, little is known about how patients learn about ICI risks and their experiences of toxicity.

Understanding ICI toxicity from patients’ and caregivers’ perspectives is essential for providing comprehensive care and improving communication between clinicians, patients, and caregivers. Understanding how patients and caregivers learn about ICIs would inform the development of educational tools to promote shared decision-making. Characterizing patients’ perceptions and experience of toxicity would help clinicians prepare patients for potential outcomes to maximize their safety and adherence to treatment. With the increasing use of ICIs as neoadjuvant and adjuvant therapy in recent years (23–27), communicating the risks and benefits of ICI therapy effectively is of paramount importance. We therefore conducted a qualitative study to explore patients’ and caregivers’ perspectives on receiving education about ICI risks and benefits, their experiences of ICI toxicity, and patient-clinician communication about toxicity to improve education and support for patients and caregivers.

## Methods

### Study Design

We conducted a qualitative study including patients with advanced melanoma and lung cancer who were initiating or discontinuing treatment with immunotherapy and their caregivers to explore patients’ and caregivers’ experiences learning about and receiving immunotherapy. The methods and findings are presented according to the Consolidated Criteria for Reporting Qualitative Research (28). We obtained institutional ethics approval from Dana-Farber/Harvard Cancer Center (DF/HCC 18–562) and all participants provided written consent. The study was conducted in accordance with the Declaration of Helsinki.

### Participants

We included patients at a single academic medical center who were diagnosed with NSCLC or melanoma and who were receiving or had received an ICI. We identified eligible patients through oncology team meetings and chart review (a Health Insurance Portability and Accountability Act waiver was obtained). After obtaining oncologist permission, patients were approached in person to introduce the study and obtain their informed consent. We used purposive sampling to obtain a broad perspective on patients’ experiences across the treatment spectrum, including patients who had recently initiated or discontinued an ICI. We included patients ≥ 18 years of age with a diagnosis of metastatic melanoma or NSCLC within 12 weeks of initiating ICI or within 12 months of discontinuing ICI, and patients with stage III melanoma that were within 12 weeks of initiating ICI. We limited the study to participants who spoke English. We asked patients to identify their caregivers (defined as family or friends involved in their medical care) and obtained caregivers’ informed consent to participate. Patients without caregivers were eligible to participate. Some patients participated in the audio-recorded visits but did not provide interviews.

### Data collection

We collected sociodemographic data via survey and clinical data from the electronic health record. Patients were asked a demographic questions and a screening question for health literacy in a survey (29). Survey data were collected on paper or electronically using Research Electronic Data Capture (REDCap)(30).

We audio-recorded and transcribed the patients’ first oncology visit after enrollment in the study, which was either before or soon after initiating an ICI, or after ICI discontinuation. Visits ranged in length from 8–93 minutes; the median (IQR) was 27.5 (18.50–40.25).

Three authors (L.P [palliative care physician, female], R.S. [research coordinator, male], A.Z. [research coordinator, female]) conducted in-depth interviews using a semi-structured interview guide that we developed for this study. The interview questions were open-ended and asked how patients learned about ICIs and their experiences over the course of treatment, as well as their recommendations for what would be helpful to others in the same situation. Patients and caregivers were interviewed separately by telephone or in person for 12–113 minutes, the median (IQR) was 27.0 (19.5–34.0). Interviews were recorded and transcribed for analysis.

### Analysis

We used descriptive statistics to summarize survey responses and clinical data. We adapted steps from the framework approach to analyze interview transcripts, including familiarizing ourselves with the data and developing a codebook based on the interview questions and concepts observed in the review of the initial transcripts (31). Codes were derived using the inductive approach (32). The transcripts were double-coded by K.H. (research assistant, female), and A.S.F. (internal medicine resident, male) which were constantly compared and reviewed for discrepancies that were resolved. L.P. and A.S.F. then conducted a case-based analysis of the audio-recorded oncology visit transcripts to triangulate observations across sources (interviews and visit recordings), with the goal of contextualizing patient observations about clinician communication (33). We synthesized codes into themes, developed statements to articulate themes via discussion among our multidisciplinary team, and extracted a limited number of quotes and visit excerpts to illustrate themes.

## Results

### Participant characteristics

From May 2019 to June 2021, we identified 113 eligible patients and approached 93, of whom 56 provided consent to participate. Most patients declined because they were not interested in research or extra obligations beyond their cancer treatment. We conducted interviews with 42 patients and 10 caregivers; the remaining patients who provided consent contributed only surveys or an audio-recorded visit. Demographic and clinical characteristics of the patients are shown in Table 1. The median age of patients was 67 (range 38–86). Twenty-six (68.4%) were male, 26 (61.9%) had melanoma, and 16 (38.1%) had non-small cell lung cancer. Eighteen (48.6%) had a college degree or higher. Pembrolizumab was the most common treatment received by 31 patients (73.8%); 10 (23.8%) received combination immunotherapy with ipilimumab/nivolumab. Among the 20 patients (47.6%) who discontinued therapy, the reasons included disease progression, immune-related adverse events, and completion of a standard number of cycles.

### Summary of themes

We identified four themes that describe the experiences of patients and caregivers learning about and experiencing ICI toxicity: i) Patients initiating an ICI received extensive information about side effects, which some patients found overwhelming or scary and difficult to absorb; ii) Patients who were deterred by toxicity fears ultimately proceeded with treatment because of oncologist encouragement or the sense of no alternative; iii) participants found hope in the association between toxicity and ICI efficacy; iv) Caregivers helped patients navigate the deluge of information and uncertainty related to ICIs. We also summarized participant suggestions to improve the immunotherapy experience and side effect education.

The themes are described below with representative quotes from participants and excerpts from visit transcripts. The themes are also presented in Table 2 with additional quotes from patients and caregivers and excerpts of patient-clinician communication from visits. Quotes are attributed to specific participants by “Pt” for patients and “CG” for caregivers and study IDs (e.g., CG 7 is caregiver 7).

#### Theme 1: Patients initiating an ICI received extensive counseling about side effects, which caused some patients to feel overwhelmed or afraid, and patients had difficulty absorbing the information.

In oncology visit recordings, clinicians communicated complex information about ICI toxicity by providing patients and caregivers with an exhaustive recitation of risks. One patient noted, “I think this was good, but it was almost pounded into me... I spent probably a good hour with [the nurse practitioner] and she went through everything, every possible side effect that could happen, the things I’d have to look for.” The same patient reported supplementing their education with searching information online, because “it’s really hard with the doctors… they give you information but sometimes maybe a little too much to digest all at once.” (Pt 140). Others were left with only vague impressions of what they learned: “I guess there were a couple side effects that were brought up that there’s certain percent chances, which I—don’t quote me on the numbers, but somewhere like 10% chance of this, 30% chance of that… Swelling of the liver, maybe some pancreatitis, and I think that’s all I remember.” (Pt 150). Another patient who experienced toxicity expressed, “To be honest with you, I cannot remember if [the toxicity they experienced] was one of the side effects discussed. I really, honestly do not remember.” (Pt 7).

Clinician education about toxicity often included medical jargon. “The immunotherapy, what it does is it’s revving your own immune system up to recognize and attack cancer cells, but sometimes, it inadvertently it kind of causes misguided *inflammation* to any other system in the body, so that’s why we say always just let us know change from your *baseline*.” (Pt 150 oncology visit recording). Patients’ comments revealed their interpretation of explanations with jargon: “Everybody I spoke with was like… call us if anything seems abnormal and we have a better chance of dealing with it, except with the glands, because those just, I guess, *burn up*, or whatever you call it.” (Pt 142).

The toxicity counseling had an emotional impact on patients. Some patients described the experience of learning about toxicity as “shocking” or “scary”. A caregiver stated: “We were concerned with side effects and the way that they’re listed on the computer or on the advertisements, it’s scary to see that.” (CG 7) Another patient stated that learning about ICI toxicity was “kind of like watching a commercial on TV for psoriasis. You don’t want to listen to the last part of the ad because it could get rid of your psoriasis but also kill you.” (Pt 142)

#### Theme 2: Patients who were deterred by fear of toxicity ultimately proceeded with treatment because of oncologist encouragement or the sense of no alternative.

Several patients reported that they found comfort in the oncology team emphasizing how infrequently serious side effects occur and how the team is prepared to manage whatever toxicity they might experience. A patient noted, “They went into the detail, but then kind of said, ‘Look, we can take care of those things as they come up, and we would use steroids.’” (Pt 42). In the audio recorded visit, this was corroborated by clinician reassurance and reframing of the risks they had just presented as manageable and rare (Table 2).

By contrast, some patients proceeded with ICIs despite their fear of toxicity because they felt that they had no alternative. One patient said, “Even when we went there, my wife said to me, “Are you sure you want to start this?” And I basically told her, I said, ‘Well, I don’t really have a choice at this point.” (Pt 141). Another patient said, “You don’t really have a lot of choices because all the information is on one side of the field. I mean, the doctors and the medical staff have everything they know as well as a ton of experience. Me? I just know I’m sick, and I need someone to help me not be sick. So, whatever they said is what my option was going to be.” (Pt 35)

#### Theme 3: The association between immunotherapy toxicity and efficacy was a source of hope.

Patients were often disappointed when they had to discontinue ICI treatment due to toxicity. However, patients sometimes found comfort or motivation in the positive implication of side effects as signs of treatment heading in the right direction. “Just two immunotherapy sessions in, and I already developed the side effects. So it’s already inflamed them all, and it’s already fighting them.” (Pt 22).

These comments echoed counseling patients received in audio-recorded oncology visits (Table 2), in which clinicians introduced and reinforced the association between toxicity and efficacy as another source of reassurance and hope: “On the combination treatment, severe side effects have been [seen] in over half of patients… I don’t say that to scare you. In fact, it’s actually maybe a good thing that if we rev your immune system up so much that it’s going out and attacking the wrong things, and causing problems, it’s probably annoying your melanoma just as much.” (Pt 22 oncology visit)

One patient even wished for side effects: “There’s a few side effects that would be great if I got, like vitiligo or something, and I hope every day, I wish to find them. I had a rash. That made me happy because maybe a rash can be a good side effect that might say that the immunotherapy is working.” (Pt 10).

#### Theme 4: Caregivers played a critical role in helping patients navigate the deluge of information and uncertainty related to ICIs and served as their advocates in oncology visits.

Participants shared that they had difficulty processing information during oncology visits and relied on caregivers to absorb information. One patient said, “I always bring somebody with me, because whenever I go to the doctor I just sort of blank out half the time. And it’s always good to have somebody with me that can remember what was said or—I’m still in a state of shock with the whole thing somewhat. Sometimes I’m there physically but I’m not there mentally, I guess.” (Pt 6). Another patient reported: “Everything’s such a blur at first… That’s why I had my husband and my daughter with me because they would have to explain everything to me afterwards. It was like, ‘You know what? I don’t even remember.’ And I think I was still in shock… you hear cancer and of course, it scares the [expletive] out of you.” (Pt 7).

In oncology visits, caregivers’ comments corroborated patients’ characterization of caregivers as their advocates. They shared their observations about patients’ symptoms and asked questions about ICI risks. For example, a caregiver asked, “If we’re in the hospital with the side effects… after the second dose… we’re not thinking like, ‘Oh, this is not working?’” (Pt 10) Another caregiver asked the oncologist a question during a scan review: “So that means that, in spite of the treatment, he’s metastasized?” and asked if the scans showed any lesions in the ribs because of new discomfort the patient was experiencing (Pt 15).

#### Participant recommendations to improve immunotherapy education and experience

Patients and caregivers offered ideas of how to personalize education about ICI risks and benefits. One patient suggested using prior patients’ experience to get the message across: “Being able to hear about specific situations, whether they ended positively or negatively, might have helped me: ‘This person started out with feeling tired and having just kind of some sore legs, but it turned out to be x y z.’ … I need specific examples to process it fully.” (Pt 153). A caregiver suggested a platform with education for patients and caregivers. “It would be good if there was a particular website that you could look up with possible symptoms, possible outcomes, anything you can do yourself, physically, to help with the treatment.” (CG 7). Some patients and caregivers suggested providing a list of frequently asked questions: “When you’re hit with it, you’ve never heard of it… after you’ve listened, you go home, and you forget half of what you’ve heard. Sometimes it’s good just to have something that tells you exactly what it is. What the side effects are. What to expect. Questions to ask…just something to have at home to refer or to keep that you can go back and look at again.” (CG 13). Participants stressed the importance of combining patient-facing education with patient-clinician communication. One patient suggested “a multi-pronged approach to communication” that would be delivered in different formats based on technological savvy: “if the person’s 85 years old, a hand-out. If the person’s 19 years old, link to a website with more info. One-on-one communication with the doctor should happen regardless of the rest.” (Pt 40).

## Discussion

In this qualitative study of patients’ and caregivers’ experiences learning about immunotherapy, we found that emotions were central to their understanding and experiences of immunotherapy toxicity. Specifically, we found that many patients had limited ability to retain specific details from the extensive counseling they received, partly because of their feelings of being overwhelmed, in shock, or afraid of what might happen. Rather than retaining the information itself, patients had strong recollections of reassurance from the oncology team regarding the manageability of side effects, which helped them overcome their fears about toxicity. Patients also internalized oncologists’ reframing of toxicity as an indication that treatment was working and this was an additional reassurance that helped them proceed despite the uncertainty and risk. Caregivers also stepped up when patients were overwhelmed, to take in information or advocate. Finally, patients and caregivers provided valuable suggestions on how to personalize education about the experience and side effects of ICIs.

The themes we identified echo prior research with new variations introduced by a novel treatment paradigm. Prior studies have demonstrated that patients especially struggle to recall information after being presented with a difficult prognosis (34). The limited information retention that some patients in this study demonstrated is a possible explanation for our prior finding of knowledge deficits about immunotherapy risks and benefits among patients starting therapy (14). It has also been well documented that a patient’s relationship with their clinician is the strongest influence on medical decision-making (35), which we found to be especially true among patients facing uncertainty related to ICI toxicity. The problem of the uncertainty that they felt was not solved by their clinicians layering on more information, but rather by the clinicians’ reassurance and reframing of ICI risks. The finding that caregiver support is critical to processing complex information aligns with prior studies that have found that caregivers improve adherence and continuity of care (36).

Our finding that patients had a surprisingly positive outlook on toxicity was another reflection of how oncologists’ communication shapes patients’ expectations. The association between immunotherapy toxicity and response has been demonstrated in several studies (37, 38); however, patients in our study shed new light on the impact of this association on their emotional experience. Heightened somatic vigilance is common among patients with cancer, and physical symptoms may trigger worry about cancer recurrence or progression (39). Patients in our study demonstrated heightened attention as they anticipated toxicity, which may add to stress even if the potential meaning of such changes is hopeful rather than worrisome.

These findings have implications for future research and policy. In 2020, the Society for Immunotherapy of Cancer Subcommittee on Quality identified gaps in quality measures for immunotherapy (40). The committee identified patient safety as a priority and emphasized patient self-reporting of adverse events as a promising approach to improving care quality. Communication and education are essential steps toward the patient empowerment necessary for self-reporting. Future research should explore strategies to optimize patient and caregiver education about ICI risks and benefits. Even with optimal delivery, however, patient education may be insufficient to overcome barriers to timely self-reporting of side effects, such as concern about bothering clinicians or appearing ungrateful for care (41). It is incumbent on clinicians and health systems to be highly receptive and responsive to patient and caregiver contact for symptom self-reporting to work, especially given the emotional component of patients’ experience with immunotherapy that we observed. Studies of patient-reported outcome reporting among patients receiving immunotherapy should focus on implementation strategies to overcome these barriers and incorporate patient and caregiver perspectives.

We identified several opportunities for optimizing communication in clinical practice (Table 3). Our finding that patients were often emotionally overwhelmed when they received ICI education points to the need to first assess their preferences for receiving information, including the support people they need with them since we identified such a strong reliance on caregivers to process information. Furthermore, given that we identified such a pronounced emotional component of learning about immunotherapy, education strategies that center on patient and caregiver needs and attend to their emotions are in order, consistent with a humanistic approach to learning (42). For example, clinicians may involve patients in setting the educational agenda to prioritize their needs and concerns (43). This may be achieved with decision aids or tools such as question prompt lists, which are lists of suggested questions that increase patient engagement in learning about their cancer and prognosis (44).The finding that patients had difficulty retaining detailed information also highlights the need for communication strategies that involve iteratively evaluating patient understanding and tailoring education (e.g., “teach-back” methods) to improve comprehension (45) In addition, incorporating anecdotes or patient descriptors of toxicity into education about immunotherapy side effects may also boost patient engagement (46).

Patients and caregivers in our study were reassured by oncologists’ emphasis on their ability to manage side effects. Such reassurance is only possible, however, when oncologists have the knowledge and access to specialist care required to manage complex immunotherapy toxicity. ICI adoption in the United States has been uneven, with lower uptake in community hospitals and among patients with Medicaid compared to private insurance (47). Oncologist unfamiliarity with ICI toxicities and limited resources to safely care for patients is likely a factor in this disparity, as well as prohibitive cost. Efforts to improve education about ICIs must focus not only on patients but also on clinicians managing patients with cancer, including oncologists, primary care and emergency medicine clinicians, and specialists (48). To achieve equity in cancer care, it is essential that all patients have access to lifesaving, guideline-recommended therapies, including ICIs, and that clinicians have access to training and decision support to deliver them safely.

### Strengths and Limitations:

The qualitative nature of the study allowed for detailed descriptions of patients’ and caregivers’ experiences with ICI communication and side effects. A potential limitation is that we conducted the study in an academic center with a well-educated patient population from a narrow range of sociodemographic backgrounds whose experiences may be less relevant to patients in other settings. The academic medical center setting also likely contributed to our conclusions regarding the comprehensive immunotherapy counseling; there may be other patient education challenges in other settings such as non-academic community oncology practices that warrant further study. It is also possible that there was a ceiling effect of clinician regard limiting the ability of patients and caregivers to identify areas for improvement in communication and patient education.

## Conclusion

Patients and caregivers received extensive counseling about ICI toxicity, yet emotions limited their ability to retain information. They were reassured by clinicians’ ability to manage toxicity and by the hopeful association of toxicity with immunotherapy response. Participants in this study highlighted opportunities to enhance communication with patients and caregivers about ICI education and we identified several opportunities to optimize ICI education and provided recommendations. These findings may inform future research and efforts to tailor education and support for patients initiating ICIs.

## Figures and Tables

**Table 1:** Participant Characteristics

	Patients (n = 42) N (%)	Caregivers (n = 10) N (%)

**Gender**	12 (31.6%)	5 (55.6%)
Female	26 (68.4%)	4 (44.4%)
Male	4	1
Missing		

**Age**, median (range), years	67 (38–86)	77 (31–80)

**Race**	36 (94.7%)	9 (100%)
White	1 (2.6%)	0
Black or African American	4	0-
Native Hawaiian or Other Pacific Islander	4	1
Missing		

**Diagnosis**	26 (61.9%)	**Caregiver of patient with**
Melanoma	16 (38.1%)	Melanoma: 7 (70%)
NSCLC		NSCLC 3 (30%)

**Education Level**	11 (29.7%)	**Relationship to patient**
Grade 12 or GED	8 (21.6%)	Spouse: 8 (80%)
College (year 1 to 3/ technical school)	8 (21.6%)	Sibling: 1 (10%)
College (graduate)	7 (18.9%)	Other family member: 1 (10%)
Master’s degree	3 (8.1%)	
Doctorate/Medical degree/Law degree	5	
Missing		

**Self-reported Health Literacy**	19 (48.7%)	
Extremely confident	10 (25.6%)	
Quite a bit	6 (15.4%)	
Somewhat	4 (10.3%)	
A little bit	0 (0.0%)	
Not at all	3	
Missing		

**Type of ICI**	31 (73.8%)	
Pembrolizumab	1 (2.4%)	
Nivolumab	10 (23.8%)	
Ipilimumab/Nivolumab		

**Reason for ICI discontinuation**	N = 20	
Progressive disease	9/20 (45.0%)	
Immune-related adverse event	10/20 (50.0%)	
Other	1/20 (5.0%)	

**Table 2:** Themes, Quotes, Visit Excerpts, and Patient Suggestions

Themes	Subthemes and example quotes from patients/caregivers	Examples from clinician visits
*Patients initiating an ICI received extensive information about side effects, which some patients found overwhelming or scary and difficult to absorb*	*Patients likened side effect counseling to listening to a drug commercial.* It makes you, obviously, wish you never had cancer in the first place to have to go through with that… it was very shocking. But then you think about just listening to anything on TV for an ad for any kind of pharmaceutical, there’s a million and a half side effects. So I guess it’s not uncommon. (Pt 145) *Emotions affected patients’ ability to receive information.* I get overloaded by too much information, and I’d rather not think ahead… I tend not to remember things if it’s anywhere close to being upsetting… I’m not stupid. I have a PhD. I can understand science, but when it comes to the cancer, if it’s too close, I start to phase out. (Pt 10)	*Clinicians used medical jargon such as “inflammation” that may not be comprehensible to patients.* Clinician: Yes. So it can cause misguided *inflammation* or irritation to any system. So it can be your brain, your skin, your lungs, your liver, your kidneys, your joints, your muscles– literally every system. (Pt 145) Clinician: One important thing is that the immune therapy can cause a host of *immune-related side effects.* Usually not right away, not within the first couple days, but over a few weeks to a few months, some patients will develop symptoms of an *immune sort of activation* that’s basically affecting normal *tissue*. (Pt 30)
*Patients who were deterred by toxicity fears ultimately proceeded with treatment because of oncologist encouragement or the sense of no alternative*	*The oncology team provided assurance about the benefit of treatment.* Well, the oncologist … was a little more convincing in that the outcomes– she was pretty sure the outcome of treatment would outweigh any side effects, and she also felt that [patient]’s general good health, beside the melanoma– that comes into effect as well. (Cg 7) It was very clear what the side effects could possibly be. There was a host of them, .... But being made aware of what the most common ones, that was something I appreciated coming from the oncology team was their clarity of the most common side effects versus the ones that are in a very small group, less than 5% or less than 1%. That kind of helps put your mind at ease when you’re trying to make a decision. (Pt 149) *Some patients perceived that treatment was the only viable option.* But I walked away from that with my last question to [the oncologist]; I said, ‘Based on all these side effects, what if I do nothing?’ And he was very candid and polite, and he said, ‘Not right away, but you’ll die.’ (Pt 142) I think it wouldn’t have changed anything how I felt. I mean, I still would’ve gone ahead and done everything. (Pt 7)	*The oncology team provided assurance about their ability to manage side effects* Clinician: Most people feel very well and have a very good quality of life on treatment. And then there’s a smaller percentage of people that can get really sick. But if you do, we’ll get you through it. We’ll treat the side effects. (Pt 16) *Oncologists emphasized the risks versus benefits of treatment.* Caregiver: So I have a question.. when we’re talking about risk versus benefits, and where this is purely preventative for him, is it really worth it? Because after hearing what you’re saying, his quality of life might change drastically if he gets Type I diabetes or something of the sort, and where essentially he has nothing now, is this really worth it?... Clinician: Yeah, it’s a very good question, and so it’s something that we kind of hem and haw over as well of like, “Does it make sense to give patients treatment when we’re not sure if there’s remaining cells, or do we wait and see if something re-grows?" I think the concern here is that your disease has already shown that there was obviously something lingering…. Certainly, if things come back in what we call stage IV, then many of those patients cannot be cured.” (Pt and CG 11)
*Participants found hope in the association between toxicity and ICI efficacy*	*Patients and caregivers correlated toxicity with efficacy.* And I got the bad rash, which I understand is the good one, because you can just treat it with a topical steroid. And so I got a heck of a rash, literally head to toe. And so they stopped my therapy after the third infusion, so I didn’t get the fourth one… I guess the only thing that makes me feel good is that my recovery process now, while it is just little baby steps, I’m watching what the immunotherapy is doing to me and my immune system, and I’m really way ahead of the curve. I am feeling awesome, my numbers, when they take, draw my blood, my lab results, are phenomenal. I’ve got numbers that are going in the right direction well ahead of a pace that they should be. (Pt 35)	*Clinicians explained the possible correlation between toxicity and efficacy.* Clinician: Yeah. I can show you the pictures if you want, but it’s really quite impressive what your immune system has done to the melanoma. Patient: Wow. I mean, I really got a lot of weird things from it but-- Clinician: I know. Well-- Patient: And somebody said to me, he says maybe that’s good. Maybe it’s doing a number. Clinician: You’re right, actually. There’s some evidence that people who get some side effects like this have a better chance of an immune response. The immune system is obviously very angry right now and so it’s attacked the melanoma. Patient: Well, that’s fabulous. (Pt 13) Clinician: That’s a good question. So at any point, it’s possible. The peak time that we would see side effects is after the second or third dose. We’ve seen side effects after one dose, but that’s less likely, just because side effects only happen when the immune system gets revved up and we don’t expect it to be fully revved up after just one dose. And it may never happen. You might do a whole year, feel fine–and that doesn’t mean that the drug’s not working if you don’t have a side effect, which we get a lot. Because that makes sense to think that way, like, “If I’m not having some type of sign, does that mean my immune system’s not revved up?” And it could be, but just not irritating the healthy parts of your body. (Pt 32)
*Caregivers helped patients navigate the deluge of information and uncertainty related to ICIs*	*Caregivers helped patients process information when they were overwhelmed* I feel very lucky that (patient’s husband) and I have a really good relationship…I have him to bounce everything off of and reality test. I do a lot of reality testing, like, “This is what’s happening. What do you think? Look at this. What do you think?” …if I were a single person who didn’t have a partner and didn’t have a close social network to bounce this stuff off of on an immediate everyday kind of way, I think I would be a mess. (Pt 10) And so I’ve been fortunate to have my family there to listen to a lot of the information because it just frankly kind of goes over my head and just kind of– I just can’t absorb everything because you’re just kind of overwhelmed at the time, just the thought of, “What? I’ve got cancer?” (Pt 30)	*Caregivers helped patients process information when they were overwhelmed* Clinician: This whole packet is for you. Patient: Okay. Clinician: Absolutely. Yeah, and whether or not you want to read through that is really up to you. We want you to have it as a resource available for you. Patient: My wife will read through it. Clinician: So you know what? Today is so overwhelming as it is, that my suggestion is read it at another time. Family members, caregivers always like to read that information. (Pt 32) *Caregivers helped with symptom monitoring.* Clinician: We are experts on the drugs, and so we can get people through it safely if there’s good communication. You’re never bothering us. You’re never annoying us. You’re never complaining. We call it accurately reporting. And luckily, you’re here with somebody else who can hear this and most of the time, the spouse is the one ratting the patient out about like, “Oh, you really need to call,” or calling and saying, “I’m only calling because my wife said I had to,” and we’re like, “Well, she was right.” (Pt & CG 11)

**Table 3:** Opportunities to optimize communication about ICI risks and benefits

Communication concept	Opportunities for optimization and patient/caregiver suggestions from study findings	Recommendation for clinicians to optimize ICI toxicity counseling
Optimizing patient-centeredness using core communication skills* (49)	• Patients may be in shock related to their diagnosis or progression and have difficulty absorbing education about ICIs • Patients rely on caregivers to help them take in information about their treatment plan and advocate for them	• Gauge the patient’s information preferences: how, when, with whom to receive ICI education • Collaborate with patients and caregivers to set an agenda for discussions about treatment options including ICIs and elicit patient/caregiver questions and concerns • Understand and respond to patient and caregiver emotions related to cancer that may be evoked by ICI counseling • Acknowledge the role of caregiver and actively incorporate caregiver in discussion
Delivering an effective message** (50)	• Patients have difficulty recalling complex information about ICI risks and benefits, especially if it is presented verbally	• Provide accurate and understandable information: review the pros and cons of ICIs in an easy-to-understand format, such as a decision aid, to support patients in understanding their treatment and making appropriate decisions; Minimize use of medical jargon and words not easily comprehensible by patients and caregivers • Promote the credibility of the information by affirming patients’ values and including a narrative: Personalize education with stories of patients who have experienced ICI toxicity to increase engagement. • Check for understanding: use teach-back method to ensure patients and caregivers understand the ICI risks and benefits • Educate patients about the correlation between toxicity and efficacy; Emphasize how and when ICI effect will be assessed and the range of possible outcomes
Providing information about all treatment options* (49)	• Some patients felt they had no alternative to treatment despite being worried about ICI risks	• Provide information about all treatment options, including alternatives to ICI treatment, as well as justification for recommending an ICI • Emphasize patients’ agency in making decisions about ICIs; explore what is motivating them to pursue treatment and their goals for treatment to match a recommendation to their goals

* Communication recommendations adapted from the American Society of Clinical Oncology Consensus Guideline on Patient-Clinician Communication by Gilligan et al, JCO (2017);

** Communication recommendations adapted from “Delivering Effective Messages in the Patient-Clinician Encounter” by Cappella and Street, JAMA (2024).
